# Body height and the excess cancer risk in men

**DOI:** 10.1002/ijc.70108

**Published:** 2025-08-26

**Authors:** Cecilia Radkiewicz, Gustaf Edgren, Arvid Sjölander, Emelie Benyi, Mats Lambe, Paul W. Dickman, Lars Sävendahl

**Affiliations:** ^1^ Department of Medical Epidemiology and Biostatistics Karolinska Institutet Stockholm Sweden; ^2^ Clinical Epidemiology Unit, Department of Medicine Solna Karolinska Institutet Stockholm Sweden; ^3^ Department of Cardiology Södersjukhuset Stockholm Sweden; ^4^ Division of Pediatric Endocrinology, Department of Women's and Children's Health (KBH) Karolinska Institutet Stockholm Sweden; ^5^ Pediatric Endocrinology Unit Karolinska University Hospital Stockholm Sweden

**Keywords:** cancer risk, cohort study, height, mediation analysis, sex differences

## Abstract

Men have a higher risk than women for most cancers affecting both sexes. Since taller stature is associated with increased cancer risk and men are, on average, taller than women, we investigated to what extent adult body height, as a proxy for stem cell number, explains the elevated cancer risk in men. This population‐based cohort study linked adult height information from Swedish conscripts, mothers, and passports to the National Cancer and Cause of Death Registers (1960–2011). We used mediation survival analysis to estimate the proportion of the association between male sex and site‐specific cancer risk mediated by adult height, our main outcome. Statistical significance was assessed using two‐sided tests with a .05 significance level. Among 6,156,659 adults, we observed 285,778 non‐sex‐specific cancer cases. Male sex was significantly associated with cancer risk at 33 of 39 sites, and greater height with increased risk at 27 of 39 sites. Height mediated 0.5%–100% of the excess male cancer risk, with the highest proportions for salivary gland cancer, colon cancer, melanoma, and acute myeloid leukemia. The effects of height and its mediated effect were most consistent for malignancies with weak or no established environmental risk factors. These findings indicate that a substantial proportion of the excess cancer risk in men may be explained by height. This highlights the role of stochastic biological processes linked to height, as well as genetic and early‐life determinants of height, in contributing to sex differences in cancer risk, beyond influences of adult lifestyle and environmental exposures.

## Graphical abstract

What's new?

In cancers that affect both sexes, men usually have a higher risk than women. While this is often attributed to behavioral factors, such as exposure to environmental carcinogens, there may be an intrinsic biological mechanism involved. Tall stature has been associated with increased cancer risk. Here, the authors report the results of a cohort study evaluating the association between body height and cancer risk for 39 different malignancies. They found the height differences accounted for much of the excess cancer risk in men, particularly in cancers with few known environmental risk factors.

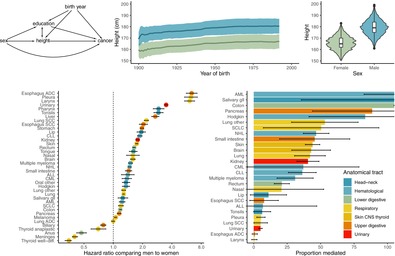

**Abbreviations**
ADC: adenocarcinoma
ALL: acute lymphocytic leukemia
AML: acute myeloid leukemia
CI: confidence interval
CLL: chronic lymphocytic leukemia
CML: chronic myeloid leukemia
gll: glands
HR: hazard ratio
ICD: International Classification of Diseases
NCR: National Cancer Register
NHL: non‐Hodgkin lymphoma
PE: proportion explained
SCC: squamous cell carcinoma
SCLC: small cell lung cancer
well‐diff.: well‐differentiated

## 1 INTRODUCTION

Men have a higher risk than women for most malignancies affecting both sexes.^1^, ^2^ This has often been attributed to greater exposure to environmental and lifestyle carcinogens, such as smoking and alcohol consumption.^3^ However, the consistency of the male predominance across calendar time periods, age groups, geographical regions, and cancers with weak or no known links to external carcinogens suggests that intrinsic biological mechanisms may also play a role. Cancer develops through the accumulation of somatic mutations in proto‐oncogenes, a process closely related to stem cell number and turnover rate within tissue.^4^, ^5^, ^6^ It has been proposed that a majority of cancer cases arise from randomly acquired deleterious mutations, a process that is modulated by carcinogen exposure and hereditary factors.^4^, ^5^, ^7^ Tall stature is associated with an increased risk of most cancer types in both men and women, except those strongly linked to smoking, such as head–neck and lung cancer.^8^, ^9^, ^10^, ^11^ Adult height is determined by genetic predisposition and external factors, including nutrition and disease during critical growth periods in utero, childhood, and adolescence.^12^, ^13^ The taller average adult stature observed in men is, at least partly, attributable to gene dosage effects on the sex chromosomes.^14^ Shared or interacting risk factors between attained height and cancer risk are plausible.^13^, ^15^, ^16^ Most previous studies examining height and cancer risk have been limited by small sample sizes or inconsistent findings.^8^, ^9^, ^10^ To our knowledge, none have been powered to use mediation analysis to study the relationship between sex and height across a complete range of non‐sex‐specific cancer sites.^17^, ^18^, ^19^

We conducted the largest cohort study to date examining the hypothesis that taller body stature contributes to the excess cancer risk observed in men, including 39 types of malignancies affecting both sexes, and using modern mediation survival analysis techniques.

## 2 MATERIALS AND METHODS

### 2.1 Data sources and study design

We conducted a population‐based cohort study including all Swedish residents with recorded adult height (≥18 years), as documented in the Swedish Conscription Register, the National Medical Birth Register, or from issued Swedish passports. Individual‐level data linkage was achieved using the Swedish personal identity number.^20^ The Conscription Register provided height measures for predominately male recruits summoned between 1960 and 2010. The Medical Birth Register included measurements for mothers enrolled in public antenatal care from 1980 to 2011. Height data from Swedish passports issued between 1991 and 2011 were obtained from the Swedish Police Authority. For individuals with multiple height records, the most frequent measurement was used; if the records were dissimilar, the tallest measurement was chosen. Measured heights from the Medical Birth and Conscription Registers were prioritized over self‐reported heights from passports. Individuals with heights outside the range of 120–220 cm were excluded.

Cancer outcomes were identified through the Swedish population‐based National Cancer Register (NCR), to which reporting is legally mandated for both clinical and pathological departments, ensuring a very high completeness, exceeding 95%.^21^ We included non‐sex‐specific incident malignancies confirmed by pathology and/or cytology and diagnosed between 1960 and 2011. Subsequent registrations of the same cancer type were excluded. The NCR records cancer cases using the currently recommended International Classification of Diseases (ICD) system for anatomical site and morphology.^21^ To facilitate time trend analyses, these classifications are translated to previous ICD versions. Details on cancer classification are provided in Table S1, Supporting Information. We grouped cancers by anatomical site using ICD‐7 and, when applicable, by histology according to the WHO Histological Classification of Neoplasms.^22^ ICD‐8 (introduced in 1980) and ICD‐Oncology‐2 (introduced in 1993) were required to further classify leukemia and lung cancer subtypes, respectively.^23^, ^24^ Brain cancer classifications included only WHO grade III–IV malignant neoplasms of the nervous system.

The National Cause of Death Register provides information on the date and cause of death, while the Total Population Register includes continuously updated data on emigration.^20^ Educational attainment was obtained from the Longitudinal Integrated Database for Health Insurance and Labor Market Studies and categorized into three levels: elementary (≤9 years), high school (10–12 years), and university (≥13 years).

### 2.2 Statistical analysis

Follow‐up time was calculated from the date of the first adult height measurement to the date of cancer diagnosis, death, emigration, or the end of follow‐up (December 31, 2011), whichever occurred first. Separate models were run for each cancer type. Cox proportional hazards regression models were used to estimate male‐to‐female hazard ratios (HR), initially without adjusting for height and subsequently including height to assess its impact on the HR estimates. Similarly, Cox models were used to estimate the cancer site‐specific HR per 10 cm increase in height, for men and women combined, as well as stratified by sex. All models were adjusted for year of birth and educational level.

#### 2.2.1 Statistical analysis: Mediation analysis

**FIGURE 1:**
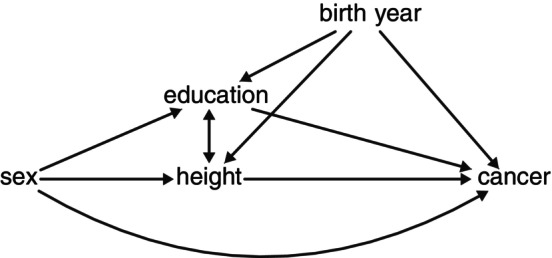
Directed acyclic graph explaining the relationship between the exposure (sex), outcome (time to cancer diagnosis), mediator (height), and measured confounders (education and birth year).

In a subsequent step, causal mediation analysis was used to quantify the extent to which body height (mediator) explains the association between male sex (exposure) and cancer risk (outcome), assuming that both exposure and mediator remain constant throughout follow‐up (adult life; Figure 1). For details, see Methods section in Data S1.

A counterfactual‐based approach was used to identify and quantify the total exposure effect and the indirect (mediated) exposure effect.^25^ The total effect represents the absolute difference in cancer‐free survival if individuals were men versus women. The indirect effect represents the hypothetical absolute difference in cancer‐free survival between men with their factual (male) height and men with a counterfactual height distribution corresponding to that of women. The proportion explained by mediation (PE) was calculated as the ratio of the indirect effect to the total effect.^25^, ^26^, ^27^ The PE can exceed 100% when the indirect effect is larger than the total effect, provided both are positive, and it can become negative when the indirect effect is positive but the total effect is negative (Figure S1). The PE may be approximated by a simple function of standard regression coefficients.^28^ This requires the mediator to fit a linear regression model and the outcome to be rare and follow a Cox proportional hazards model with no exposure‐mediator interactions.^28^ The proportional hazards assumption was visually assessed using Schoenfeld residual plots and was found to be reasonably satisfied for the majority of the 28 cancer types included in the mediation analysis (Figure S2).

All tests of statistical significance were two‐sided, with a *p*‐value threshold of .05. Stata Intercooled version 15.0 (StataCorp LP) was used for all data cleaning and analysis, except for the mediation analyses, which were performed using a user‐written function in R v.3.5.1 (R Foundation for Statistical Computing, Vienna, Austria), as described by Sjölander.^28^

## 3 RESULTS

**FIGURE 2:**
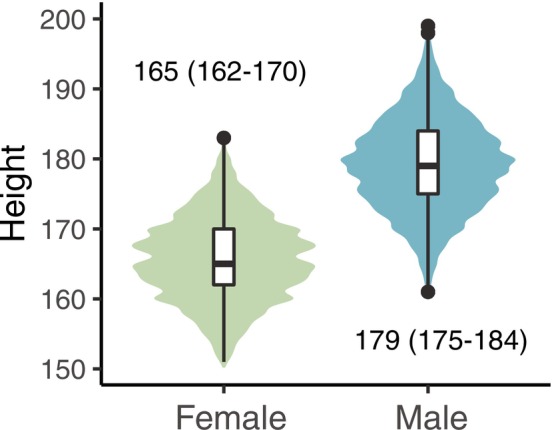
Violin plot illustrating the height distribution, median and interquartile range in cm, in women and men.

Among 6,156,659 individuals (3,133,783 men, 3,022,876 women) with a recorded adult body height, we identified 285,778 non‐sex‐specific cancer cases (164,237 in men, 121,541 in women) over 117,081,452 person‐years of follow‐up. The mean adult height was 165 and 179 cm in women and men, respectively (Figure 2), and increased over birth year in both sexes (Figure S3). Mean height increased with educational level in both sexes, a finding that was consistent over birth year (results not presented).

**FIGURE 3:**
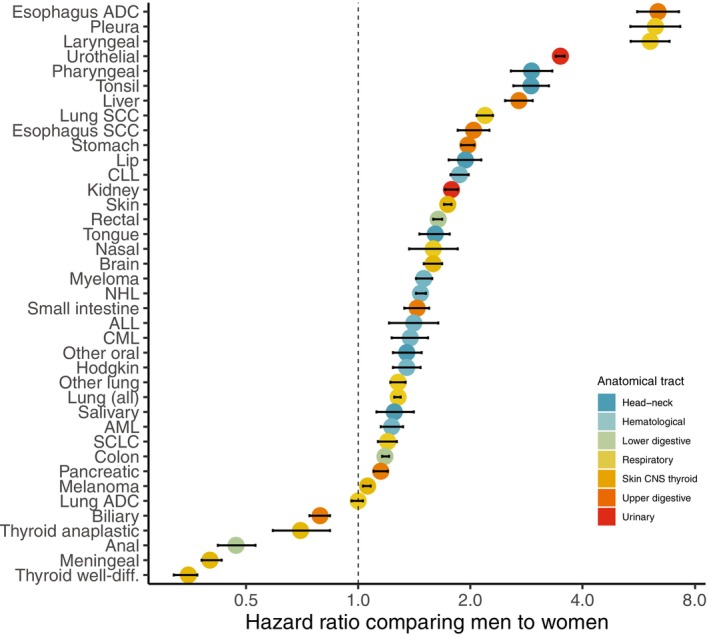
Male‐to‐female hazard ratio and 95% confidence intervals (bars), adjusted for birth year and educational level, for all investigated cancer sites, on a logarithmic scale. ADC: adenocarcinoma; ALL: acute lymphocytic leukemia; AML: acute myeloid leukemia; CLL: chronic lymphocytic leukemia; CML: chronic myeloid leukemia; gll: glands; NHL: non‐Hodgkin lymphoma; SCC: squamous cell carcinoma; SCLC: small cell lung cancer; well‐diff.: well‐differentiated.

Men had a statistically significant increased risk for the majority of the investigated cancer sites (33 of 39) compared to women (Table 1 and Figure 3). The largest male excess cancer risks (HR >2) were observed in malignancies strongly linked to smoking and/or alcohol consumption (e.g., pharyngeal, tonsil, esophageal, liver, laryngeal, and squamous cell lung cancer) and/or occupational carcinogens (e.g., urothelial cancer and pleural mesothelioma). Six cancers were more common in women: biliary, anal, meningeal, and thyroid cancer (both subtypes), as well as lung adenocarcinoma, and were therefore excluded from the mediation analyses.

**TABLE 1:** Numbers (*n*) and percentages (%) of men and women diagnosed with cancer and included in the time‐to‐event analysis. Male‐to‐female hazard ratios (HR) additionally adjusted for height (aHR), as well as HR per 10 cm increase in body height for both sexes combined, and separately for men and women. All HR estimates are adjusted for birth year and educational level, and presented with 95% confidence intervals (CI).

Anatomical tract	Cancer site	Men	Women	Male‐to‐female	Male‐to‐female	Total	Men	Women
		*n* (%)	*n* (%)	HR (95% CI)	aHR (95% CI)	HR/10 cm (95% CI)	HR/10 cm (95% CI)	HR/10 cm (95% CI)
Head–neck	1 Lip	1048 (62.9)	619 (37.1)	1.94 (1.75, 2.14)	1.79 (1.54, 2.07)	1.06 (0.98–1.15)	1.00 (0.91–1.10)	1.22 (1.06–1.40)
	2 Tongue	1167 (60.8)	751 (39.2)	1.61 (1.46, 1.76)	1.64 (1.43, 1.88)	0.98 (0.92–1.06)	1.01 (0.92–1.10)	0.94 (0.83–1.07)
	3 Salivary gll	657 (54.9)	539 (45.1)	1.25 (1.12, 1.41)	0.93 (0.78, 1.10)	1.25 (1.14–1.37)	1.18 (1.05–1.34)	1.38 (1.19–1.59)
	4 Oral other	1140 (55.7)	906 (44.3)	1.35 (1.24, 1.48)	1.41 (1.24, 1.60)	0.97 (0.90–1.04)	0.91 (0.83–1.00)	1.07 (0.95–1.20)
	5 Pharynx	920 (73.9)	325 (26.1)	2.92 (2.57, 3.32)	3.32 (2.79, 3.95)	0.91 (0.83–1.00)	0.90 (0.82–1.00)	0.94 (0.78–1.13)
	6 Tonsils	1215 (74.2)	422 (25.8)	2.91 (2.61, 3.25)	2.55 (2.19, 2.97)	1.10 (1.02–1.19)	1.10 (1.01–1.20)	1.11 (0.94–1.31)
Upper digestive	7 Esophagus ADC	1561 (85.1)	274 (14.9)	6.38 (5.61, 7.26)	6.03 (5.13, 7.09)	1.04 (0.97–1.12)	1.06 (0.98–1.15)	0.93 (0.76–1.15)
	8 Esophagus SCC	1189 (64.6)	651 (35.4)	2.04 (1.85, 2.25)	1.90 (1.65, 2.18)	1.06 (0.98–1.14)	1.01 (0.92–1.10)	1.18 (1.03–1.35)
	9 Stomach	5988 (63.4)	3452 (36.6)	1.97 (1.89, 2.05)	2.13 (2.01, 2.26)	0.94 (0.91–0.97)	0.94 (0.90–0.97)	0.96 (0.91–1.02)
	10 Liver	1827 (70.9)	751 (29.1)	2.70 (2.48, 2.94)	2.82 (2.50, 3.18)	0.97 (0.91–1.03)	0.97 (0.90–1.04)	0.97 (0.85–1.10)
	11 Biliary	1689 (41.8)	2354 (58.2)	0.79 (0.74, 0.84)	0.66 (0.60, 0.72)	1.14 (1.09–1.20)	1.15 (1.07–1.24)	1.13 (1.06–1.22)
	12 Pancreas	4475 (51.5)	4220 (48.5)	1.15 (1.10, 1.20)	1.00 (0.94, 1.07)	1.11 (1.07–1.15)	1.07 (1.02–1.12)	1.16 (1.10–1.22)
	13 Small intestine	1524 (57.1)	1144 (42.9)	1.44 (1.33, 1.55)	1.19 (1.07, 1.34)	1.15 (1.08–1.22)	1.14 (1.05–1.23)	1.16 (1.05–1.29)
Lower digestive	14 Colon	19,236 (51.3)	18,274 (48.7)	1.18 (1.16, 1.21)	0.95 (0.92, 0.98)	1.18 (1.16–1.20)	1.14 (1.11–1.16)	1.24 (1.21–1.27)
	15 Rectum	12,363 (59.6)	8390 (40.4)	1.64 (1.59, 1.68)	1.44 (1.38, 1.50)	1.10 (1.08–1.13)	1.09 (1.06–1.12)	1.13 (1.09–1.17)
	16 Anus	449 (30.5)	1021 (69.5)	0.47 (0.42, 0.53)	0.36 (0.31, 0.42)	1.22 (1.12–1.33)	1.14 (0.98–1.31)	1.28 (1.15–1.42)
Respiratory	17 Nasal	434 (60.0)	289 (40.0)	1.59 (1.37, 1.85)	1.38 (1.11, 1.72)	1.11 (0.99–1.25)	1.13 (0.97–1.31)	1.08 (0.89–1.33)
	18 Larynx	1772 (84.8)	318 (15.2)	6.08 (5.39, 6.85)	5.94 (5.11, 6.91)	1.02 (0.95–1.09)	1.00 (0.93–1.08)	1.12 (0.93–1.36)
	19 Lung	17,049 (53.7)	14,686 (46.3)	1.28 (1.25, 1.30)	1.14 (1.10, 1.18)	1.09 (1.07–1.11)	1.04 (1.02–1.07)	1.17 (1.14–1.21)
	20 Lung SCC	4316 (65.8)	2242 (34.2)	2.19 (2.08, 2.30)	2.07 (1.92, 2.23)	1.04 (1.00–1.09)	1.02 (0.97–1.07)	1.11 (1.03–1.20)
	21 Lung ADC	6345 (47.5)	7001 (52.5)	1.00 (0.96, 1.03)	0.87 (0.83, 0.92)	1.11 (1.07–1.14)	1.05 (1.01–1.10)	1.18 (1.13–1.23)
	22 SCLC	2363 (52.1)	2169 (47.9)	1.20 (1.13, 1.27)	1.08 (0.99, 1.18)	1.08 (1.03–1.14)	1.02 (0.95–1.09)	1.19 (1.10–1.28)
	23 Lung other	3983 (53.9)	3406 (46.1)	1.28 (1.22, 1.34)	1.12 (1.04, 1.19)	1.11 (1.07–1.15)	1.07 (1.02–1.13)	1.18 (1.11–1.25)
	24 Pleura	1074 (84.9)	191 (15.1)	6.28 (5.38, 7.32)	4.92 (4.05, 5.98)	1.20 (1.10–1.32)	1.17 (1.06–1.29)	1.50 (1.17–1.93)
Urinary	25 Urinary	20,316 (75.2)	6684 (24.8)	3.49 (3.39, 3.58)	3.09 (2.98, 3.21)	1.10 (1.07–1.12)	1.09 (1.07–1.12)	1.12 (1.07–1.17)
	26 Kidney	6405 (62.4)	3856 (37.6)	1.78 (1.71, 1.85)	1.35 (1.27, 1.43)	1.23 (1.19–1.27)	1.23 (1.18–1.28)	1.22 (1.15–1.29)
Skin, CNS, thyroid	27 Melanoma	15,392 (51.1)	14,721 (48.9)	1.06 (1.03, 1.08)	0.73 (0.71, 0.76)	1.31 (1.29–1.34)	1.29 (1.26–1.33)	1.36 (1.33–1.40)
	28 Skin	17,232 (59.3)	11,824 (40.7)	1.74 (1.70, 1.78)	1.31 (1.26, 1.35)	1.24 (1.22–1.27)	1.22 (1.19–1.25)	1.29 (1.25–1.33)
	29 Brain	3229 (61.9)	1988 (38.1)	1.59 (1.50, 1.68)	1.26 (1.16, 1.37)	1.19 (1.14–1.24)	1.18 (1.12–1.25)	1.18 (1.10–1.28)
	30 Meninges	1728 (28.9)	4242 (71.1)	0.40 (0.38, 0.43)	0.39 (0.36, 0.42)	1.03 (0.99–1.08)	1.04 (0.96–1.12)	1.03 (0.98–1.09)
	31 Thyroid well‐diff.	1154 (28.1)	2956 (71.9)	0.35 (0.32, 0.37)	0.27 (0.25, 0.30)	1.20 (1.14–1.26)	1.23 (1.13–1.35)	1.19 (1.12–1.27)
	32 Thyroid anaplastic	206 (41.1)	295 (58.9)	0.70 (0.59, 0.84)	0.51 (0.39, 0.67)	1.26 (1.09–1.46)	1.30 (1.05–1.62)	1.22 (1.00–1.48)
Hematologic	33 NHL	10,353 (57.8)	7544 (42.2)	1.47 (1.43, 1.52)	1.21 (1.16, 1.26)	1.16 (1.13–1.19)	1.15 (1.11–1.18)	1.19 (1.14–1.23)
	34 CLL	3198 (62.7)	1906 (37.3)	1.87 (1.77, 1.98)	1.43 (1.32, 1.56)	1.22 (1.17–1.28)	1.22 (1.15–1.29)	1.24 (1.15–1.34)
	35 Hodgkin lymphoma	1584 (64.2)	885 (35.8)	1.35 (1.24, 1.47)	1.03 (0.92, 1.17)	1.21 (1.14–1.29)	1.20 (1.12–1.30)	1.24 (1.11–1.38)
	36 Multiple myeloma	3719 (57.4)	2759 (42.6)	1.50 (1.43, 1.58)	1.29 (1.20, 1.39)	1.12 (1.07–1.16)	1.11 (1.06–1.17)	1.13 (1.06–1.20)
	37 ALL	459 (61.8)	284 (38.2)	1.41 (1.21, 1.64)	1.34 (1.08, 1.67)	1.04 (0.92–1.16)	1.09 (0.95–1.25)	0.94 (0.77–1.15)
	38 AML	1759 (53.7)	1518 (46.3)	1.23 (1.15, 1.32)	0.90 (0.81, 1.00)	1.27 (1.20–1.34)	1.27 (1.18–1.37)	1.27 (1.17–1.39)
	39 CML	726 (59.1)	502 (40.9)	1.38 (1.23, 1.54)	1.20 (1.02, 1.42)	1.11 (1.01–1.21)	1.11 (0.99–1.25)	1.09 (0.94–1.27)

Abbreviations: ADC, adenocarcinoma; ALL, acute lymphocytic leukemia; AML, acute myeloid leukemia; CLL, chronic lymphocytic leukemia; CML, chronic myeloid leukemia; gll, glands; NHL, non‐Hodgkin lymphoma; SCC, squamous cell carcinoma; SCLC, small cell lung cancer; well‐diff., well‐differentiated.

With the exception of tongue, other oral, pharyngeal, gastric, and liver cancer, the excess cancer risk in men was attenuated after adjusting for height. In four malignancies (cancer of the salivary glands, colon, malignant melanoma, and acute myeloid leukemia [AML]) the male excess risk was reversed, resulting in a height‐adjusted HR below 1.00. The majority of malignancies (27 of 39) were statistically significantly associated also with taller stature when both sexes were analyzed together (Table 1). For the smoking‐related malignancies (lip, other oral, esophageal squamous cell, laryngeal, all pulmonary subtypes, and pleural cancers), the association between height and cancer risk was weak or reversed in men, but consistent in women, suggesting possible effect modification by sex. The risk of tongue, oral cavity, pharynx, stomach, and liver cancer decreased with increasing body height (men and women combined); therefore, these cancers were excluded from the mediation analyses.

**FIGURE 4:**
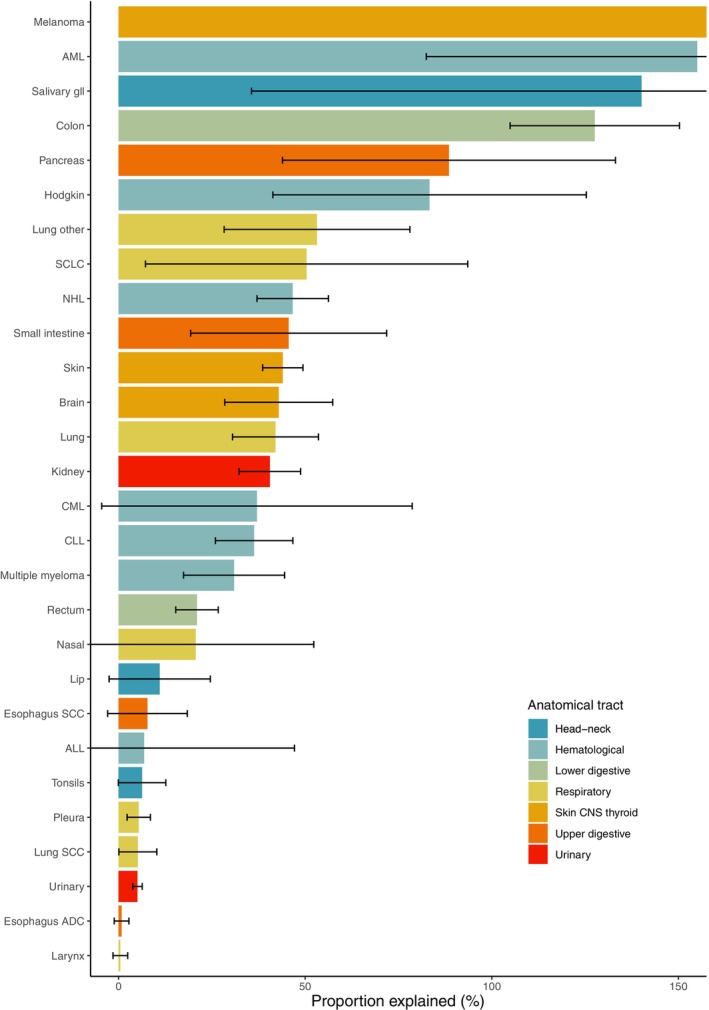
Proportion (%) of the excess cancer risk in men explained by body height, including 95% confidence intervals (bars) for all malignancies with a positive association with male sex and height, adjusted for birth year and educational level. Cancer types showing an inverse association with male sex and/or height (e.g., biliary, anal, meningeal, thyroid (both subtypes), lung ADC, tongue, oral cavity, pharynx, stomach, and liver cancers) were excluded from the mediation analysis, as negative or non‐significant total effects do not permit a meaningful interpretation of the “proportion explained.” ADC: adenocarcinoma; ALL: acute lymphocytic leukemia; AML: acute myeloid leukemia; CLL: chronic lymphocytic leukemia; CML: chronic myeloid leukemia; gll: glands; NHL: non‐Hodgkin lymphoma; SCC: squamous cell carcinoma; SCLC: small cell lung cancer.

Cancer types with an inverse association with male sex and/or height were excluded from the mediation analysis, as negative total or indirect effects prevent meaningful interpretation of the PE. Consequently, the proportion of the excess male cancer risk explained (i.e., mediated) by height was greater than 0% for all 28 malignancies included in the mediation analyses, with statistically significant mediation observed in 21 of 28 (Table 2 and Figure 4). The PE ranged from 0.5% (95% confidence interval [CI] −1.5 to 2.5%) for laryngeal cancer to over 100% for salivary gland cancer, colon cancer, malignant melanoma, and AML. Proportions exceeding 100% reflect a reversal in the direction of the sex difference after adjustment for height; that is, men had a lower cancer risk than women after accounting for height, suggesting that if men and women had the same height distribution, women would have a higher risk of these cancers. A consistent PE of around 30–50% was observed in cancers with a moderate excess risk in men, including small intestine (PE 46%, 95% CI 19–72%), non‐melanoma skin (PE 44%, 95% CI 39–49%), overall lung (PE 42%, 95% CI 31–54%), and brain cancer (PE 43%, 95% CI 28–57%), as well as in several hematological malignancies. Limited numbers of cancer events (i.e., ≤500) hampered inferences for acute lymphocytic leukemia (ALL) and chronic myeloid leukemia (CML). For the other hematological malignancies, the PE ranged from 31% (95% CI 17–44%) for multiple myeloma to 47% (95% CI 37–56%) for non‐Hodgkin (NHL) lymphoma. Sites with small (≤10%) PE values and/or values not statistically significantly different from 0% included squamous cell carcinoma of the lung, pleural, and urinary cancer. Small cancer case numbers in women (tonsil, esophageal adenocarcinoma, nasal, laryngeal cancer, and ALL) along with weak or sex‐discordant associations with height (lip, other oral, esophageal squamous cell, laryngeal, all pulmonary subtypes, and pleural cancers) may also contribute to the lack of statistical precision.

**TABLE 2:** Proportion (%) of the excess cancer risk in men explained (mediated) by body height including 95% confidence intervals (CI) for all malignancies with a positive association with male sex and height, adjusted for birth year and educational level.

Anatomical tract	Cancer site		Proportion explained (95% CI)
Head–neck	1	Lip	11 (−3 to 25)
	3	Salivary gll	140 (36–245)
	6	Tonsils	6.3 (−0.1 to 12.7)
Upper digestive	7	Esophagus ADC	0.8 (−1.2 to 2.8)
	8	Esophagus SCC	7.7 (−2.9 to 18.4)
	12	Pancreas	89 (44–133)
	13	Small intestine	46 (19–72)
Lower digestive	14	Colon	128 (105–150)
	15	Rectum	21 (15–27)
Respiratory	17	Nasal	21 (−11 to 52)
	18	Larynx	0.5 (−1.5 to 2.5)
	19	Lung	42 (31–54)
	20	Lung SCC	5.2 (0.1–10.2)
	22	SCLC	50 (7–94)
	23	Lung other	53 (28–78)
	24	Pleura	5.4 (2.3–8.5)
Urinary	25	Urinary	5.1 (3.8–6.3)
	26	Kidney	41 (32–49)
Skin, CNS, thyroid	27	Melanoma	802 (439–1166)
	28	Skin	44 (39–49)
	29	Brain	43 (28–57)
Hematologic	33	NHL	47 (37–56)
	34	CLL	36 (26–47)
	35	Hodgkin lymphoma	83 (41–125)
	36	Multiple myeloma	31 (17–44)
	37	ALL	6.9 (−33.4 to 47.1)
	38	AML	155 (82–228)
	39	CML	37 (−5 to 79)

*Note*: Cancer types showing an inverse association with male sex and/or height (e.g., biliary, anal, meningeal, thyroid [both subtypes], lung ADC, tongue, oral cavity, pharynx, stomach, and liver cancers) were excluded from the mediation analysis, as negative or non‐significant total effects do not permit a meaningful interpretation of the “proportion explained.”

Abbreviations: ADC, adenocarcinoma; ALL, acute lymphocytic leukemia; AML, acute myeloid leukemia; CLL, chronic lymphocytic leukemia; CML, chronic myeloid leukemia; gll, glands; NHL, non‐Hodgkin lymphoma; SCC, squamous cell carcinoma; SCLC, small cell lung cancer.

## 4 DISCUSSION

In this Swedish population‐based cohort study, we investigated the extent to which the higher cancer risk in men can be attributed to differences in body height, serving as a proxy for a greater number of cell divisions and the resulting excess risk of proto‐oncogene mutations. Our findings suggest that sex differences in height explain a substantial proportion of the excess cancer risk in men, particularly for cancer types with weak or no well‐established environmental risk factors.

To our knowledge, this is the largest study to date examining the relationship between sex, cancer, and body height, and the only study with a dataset large enough to assess these associations across a near‐exhaustive range of cancer types and relevant histological subtypes.^17^, ^18^, ^19^ We applied modern mediation survival analysis methods to estimate the proportion of the excess cancer risk in men attributable to body height.^28^ By cross‐linking data, our study incorporated comprehensive information on adult body height alongside high‐quality, complete registry records of cancer diagnoses, mortality, migration, and education.

Several limitations warrant discussion. First, a substantial proportion of the height estimates were self‐reported from Swedish passports, potentially introducing differential response bias, as men tend to overestimate their height.^29^ Frequent rounding of height to the nearest 10 cm (Figure 2) likely caused non‐differential misclassification, potentially diluting true associations but unlikely to generate spurious ones. Second, although our large cohort provided statistical power, we lacked individual‐level data on key cancer risk factors (e.g., smoking, alcohol consumption, diet, BMI, and occupational exposures) which were only available for a subset of the female population (e.g., smoking and BMI data for Swedish women in the National Medical Birth Register). While these factors are not formal confounders of the exposure (sex) or the mediator (attained adult height), childhood socioeconomic status may influence both attained height and adult lifestyle. To partially address this, we adjusted for educational level, a widely used proxy for socioeconomic status that also correlates with several health‐related behaviors, including smoking and diet.^13^, ^15^ While education is a useful indicator, it does not fully capture the complexity of socioeconomic and lifestyle‐related exposures.^19^ Importantly, models with and without adjustment for education yielded similar results, suggesting limited impact by these factors. Cancer‐free survival may be influenced by detection bias. However, none of the included cancers were subject to organized screening. Since men are generally less likely to seek timely medical attention, any resulting delays in diagnosis would underestimate rather than exaggerate sex differences in cancer‐free survival. Third, our methodology relies on several underlying assumptions.^28^ One key consideration is that education acts as a mediator‐outcome confounder influenced by the exposure (Figure 1). Adjusting for education reduces confounding but may also block the pathway mediated through education, potentially affecting the indirect effect and, consequently, our estimate of the proportion mediated through height.^30^ Addressing this issue requires additional assumptions, and as far as we know, no available methods exist for assessing such influence in survival outcomes.^31^ Another methodological assumption is that the outcome follows a Cox proportional hazards model, where the male‐to‐female hazard ratio remains constant over age.^28^ However, we formally tested the proportional hazards assumption and found no evidence of major violations for most cancer types included in the mediation analysis.

Our findings of a positive association between height and cancer risk align with previous studies conducted across various settings.^7^, ^8^, ^9^, ^10^, ^11^, ^32^, ^33^, ^34^, ^35^, ^36^ However, many earlier studies were underpowered due to a limited number of cancer cases,^7^, ^8^, ^9^, ^10^, ^33^ and some were restricted to one sex and/or fatal cases only.^9^, ^10^, ^32^ Studies that included additional sociodemographic covariates often reported a height‐cancer association for smoking‐related tumors only after adjusting for smoking.^8^, ^9^, ^10^, ^32^, ^33^ Congruent with our findings, the relationship between height and cancer has been particularly evident in studies of dermatological and hematological malignancies.^7^, ^9^, ^10^, ^33^, ^35^, ^37^ However, few studies have quantified the extent to which height explains the excess cancer risk in men, and those that have primarily relied on traditional mediation analyses.^17^, ^18^, ^19^ In contrast, the novel methodology in our study reduces bias from model misspecification by not depending on the confounder distribution or the effects of confounders on the mediator and outcome.^28^

This study was based on the hypothesis that the consistently higher cancer risk in men is driven by larger body size, leading to more cell divisions, increased stem cell turnover, and a higher likelihood of proto‐oncogene mutations.^4^, ^5^, ^7^, ^38^, ^39^ The concept of cumulative stem cell divisions and accumulated genetic changes over time aligns with the multi‐stage model of carcinogenesis, which explains the age‐incidence patterns of most malignancies.^6^, ^7^, ^38^, ^39^, ^40^ The observed consistency of associations between height and the risk of dermatological and hematological malignancies may be partly explained by the biological characteristics of these tissues. While the relationship between adult height, organ‐specific cell number, and stem cell number is not fully elucidated, it is plausible that taller individuals have a greater absolute number of skin and hematopoietic stem cells, due to larger body surface area and blood volume, respectively.^40^, ^41^ These tissues are also distinguished by inherently high cell turnover rates throughout life, which may increase the cumulative number of cell divisions and thereby elevate the risk of acquiring oncogenic mutations.^4^ Conversely, organs with lower rates of cell turnover or less clear scaling with height may show weaker associations. Importantly, the inconsistent association between height and cancer risk does not contradict the cell division hypothesis. Socioeconomic factors likely confound the observed “null” association between height and cancers where smoking, and to a lesser extent alcohol use or poor hygiene, are dominant risk factors.^15^, ^42^, ^43^, ^44^, ^45^ These include cancers of the head and neck,^42^ upper digestive tract,^44^ respiratory tract,^43^ and urothelial system.^45^ Except for urinary cancer, these cancer types showed weak, non‐significant, inconsistent, and/or negative associations with adult height (Table 1). In our sex‐stratified analyses, the associations between height and smoking‐related cancer risk attenuated or reversed among men, suggesting a potential interaction between lifestyle exposures and biological sex. This may be due to the complex interplay between socioeconomic status and height, which appears to be incongruent in women.^46^ Additionally, previous research has reported that smoking habits are less closely linked to socioeconomic status in Swedish women than in men.^47^

Other theories on the height‐cancer association exist, and underlying drivers may vary over time, by sex, and across cancer sites. Taller individuals may face a higher cancer burden due to increased environmental and lifestyle carcinogen exposure and/or basal metabolic rate.^48^, ^49^ Taller individuals have a larger skin surface area, which could result in greater cumulative UV exposure; however, this remains speculative and was not directly evaluated in our study. In other species, adult caloric intake is linked to cancer risk.^49^ In humans, energy expenditure generally rises with body stature; a definitive link between caloric intake and cancer risk in humans has, however, not been established.^48^, ^49^ Adult body height is a complex trait influenced by genetic, biological, and environmental factors.^12^, ^13^ Genetic variants linked to growth regulation account for 60–80% of height variability.^12^, ^13^ Height‐associated genes may influence carcinogenesis directly or indirectly through metabolism, immune function, or hormone levels. Incorporating data on family relationships and parental height could help clarify the relative contributions of genetic and environmental factors in the height‐cancer risk relationship. Growth is regulated by key hormones, including insulin‐like growth factor, growth hormone, thyroid hormones, and sex steroids, while elevated cortisol (often associated with chronic stress or illness) can suppress growth.^12^, ^13^ Insulin‐like growth factor and growth hormone are central drivers of somatic growth and have also been implicated in adult cancer risk, yet direct evidence linking their levels in childhood or adolescence to future cancer development remains limited.^50^ Estrogen may confer protection against certain adult‐onset cancers; but current evidence does not clearly support a role for estrogen levels in childhood in determining either attained adult height or long‐term cancer risk.^51^ Hormonal factors are more plausibly upstream mediators in the pathway linking sex to adult height rather than confounders of the height‐cancer association. Maternal health, nutrition, and toxin exposure during pregnancy, low birth weight, or premature birth can influence adult stature.^13^ Adequate childhood nutrition is critical for full growth potential.^12^, ^13^ Developmental malnutrition has been associated with lower levels of insulin‐like growth factors.^49^ Environmental influences on height are linked to childhood socioeconomic status, which affects long‐term health outcomes, including cancer risk.^13^, ^15^, ^16^ The steady increase in average height across birth years from 1900 to 1991, even during war and economic recessions (Figure S3), suggests that widespread malnutrition in the Swedish population in the 20th century was unlikely. In Sweden, parental health behaviors (e.g., smoking, alcohol use, and diet) likely influence attained height and future cancer risk more than childhood access to nutritious food, physical activity, or healthcare.^16^

Our findings suggest that taller body stature, likely reflecting a greater number of susceptible cells in specific organs, explains a substantial portion of the excess cancer risk observed in men compared to women. No currently known or hypothesized cancer risk factor strongly linked to height provides a better alternative explanation. These results support the view that a considerable proportion of cancers arise from stochastic processes, rather than being solely driven by hereditary factors or environmental exposures. Future research should aim to elucidate additional underlying biological mechanisms, including genetic determinants of stature and hormonal influences during key developmental periods, that may link height to cancer risk. Clinically, these insights underscore the importance of recognizing inherent biological contributions to cancer risk, while reinforcing that prevention efforts should target modifiable risk factors. Finally, this work advances the understanding of sex‐based differences in cancer susceptibility and may help refine future cancer risk predictions.

## AUTHOR CONTRIBUTIONS

**Cecilia Radkiewicz:** Conceptualization; investigation; writing – original draft; methodology; validation; visualization; formal analysis; project administration; data curation. **Gustaf Edgren:** Conceptualization; investigation; funding acquisition; methodology; validation; visualization; writing – review and editing; formal analysis; supervision; resources. **Arvid Sjölander:** Conceptualization; investigation; methodology; validation; visualization; writing – review and editing; software; formal analysis; supervision. **Emelie Benyi:** Conceptualization; writing – review and editing; project administration; data curation. **Mats Lambe:** Conceptualization; writing – review and editing; project administration; supervision; resources; funding acquisition. **Paul W. Dickman:** Conceptualization; investigation; funding acquisition; methodology; validation; writing – review and editing; formal analysis; project administration; supervision; resources. **Lars Sävendahl:** Conceptualization; writing – review and editing; project administration; resources.

## CONFLICT OF INTEREST STATEMENT

The authors declare no conflicts of interest.

## ETHICS STATEMENT

The study was conducted in accordance with the Helsinki Declaration and approved by the Ethical Review Board, Stockholm, Sweden (reference numbers 2011/1267‐31 and 2017/2145‐32). Written informed consent is not required for this type of large, observational, register‐based study.

## Supporting information


**Data S1.** Supporting Information.

## Data Availability

Aggregated data that support the study findings are available from the corresponding author upon reasonable request.
