# Supplementary material for: Body height and the excess cancer risk in men
Source: Int J Cancer. 2025 Aug 26;158(3):597–607. doi: 10.1002/ijc.70108 (PMC12670345; doi:10.1002/ijc.70108)
Supplement: Supplementary file 1 — Data S1. Supporting Information. [file IJC-158-597-s001.pdf]

SUPPLEMENTARY MATERIAL

BODY HEIGHT AND THE EXCESS CANCER RISK IN MEN

Cecilia Radkiewicz, Gustaf Edgren, Arvid Sjölander, Emelie Benyi, Mats Lambe, Paul W Dickman, Lars Sävendahl

Table of contents

Supplementary methods page 2

Supplementary Table 1 page 5

Supplementary Figure 1 page 6

Supplementary Figure 2 page 7

Supplementary Figure 3 page 9

Supplementary references page 10

Supplementary methods

Statistical analysis: mediation analysis

We used causal mediation analysis to quantify the extent to which body height mediates (i.e., explains) the association between male sex (exposure) and cancer risk (outcome), assuming neither exposure nor mediator to change over follow-up (age 18-90 years). A fundamental step in mediation analysis is to identify and quantify the total exposure effect and the indirect (i.e., mediated) exposure effect (Figure 1). A counterfactual-based approach to define the total and indirect effects was used, where $S_a(t)$ denotes the proportion of subjects who would remain cancer-free at time t, had everyone had exposure level a (a=1 for male or a=0 for female). The total exposure effect is defined as $TE(t) = S_1(t) - S_0(t)$. Further, $S_{aM_{a^*}}(t)$ denotes the proportion of subjects who would remain cancer-free at time t, had everyone had exposure level a, and the mediator had simultaneously been distributed as if everyone would have had exposure level a^* (not necessarily equal to a). The indirect effect for male sex is defined as $IE(t) = S_{1M_1}(t) - S_{1M_0}(t)$. In English, this expression is interpreted as the difference in cancer-free proportions at time t, had everyone been male ($a = 1$), contrasting the counterfactual scenario where body height is distributed as if everyone were male (M_1) to the counterfactual scenario where body height is distributed as if everyone were female (M_0). This counterfactual definition of total and indirect effects is widely accepted in statistics and epidemiology.[1-3] The proportion explained by mediation, PE(t), is defined as the indirect effect divided with the total effect:[3-5]

$$PE(t) = \frac{IE(t)}{TE(t)}$$

This expression is generally a complex function of time. However, under certain assumptions, it may be approximated by a simple function of standard regression coefficients.[6] This approximation assumes no unmeasured exposure, mediator, or outcome confounder and no mediator-outcome confounder affected by the exposure. The mediator is required to fit a linear regression model and

the outcome must be rare and follow a Cox proportional hazards model with no exposure-mediator interactions. These assumptions were considered to be satisfactorily met and the proportion explained by mediation was thus approximated as:

$$PE(t) \approx \frac{e^{\gamma_1}(e^{\beta\gamma_2} - 1)}{e^{\gamma_1+\beta\gamma_2} - 1}$$

where β is the exposure coefficient in the linear regression model for the mediator, and γ_1 and γ_2 are the exposure and mediator coefficients, respectively, from the Cox proportional hazards model for the outcome.[6] The delta method was used to obtain confidence limits for the proportion mediated.

To further aid interpretation, Supplementary Figure 1 shows the relation between the proportion explained by mediation and the total effect, when the indirect effect is equal to 1 (an arbitrary positive value). The most intuitive case is when the total effect is larger than the indirect effect. In this case, the proportion explained by mediation is positive. When the total effect is close to the indirect effect (i.e., when almost all effects is through the mediator), the proportion explained by mediation is close to 100%. When the total effect is much larger than the indirect effect, the proportion explained by mediation is close to 0%.

A less intuitive case is when the total effect is smaller than the indirect effect, while still being positive. This means that the direct (i.e., not through the mediator) effect is negative, but smaller in magnitude than the indirect effect. In this case, the proportion explained by mediation is positive, but is larger than 100%. When the total effect is close to 0 (i.e., when the direct effect is just slightly smaller in magnitude than the indirect effect), the proportion explained by mediation is large in magnitude and positive.

A perhaps even less intuitive case is when the total effect is negative. This means that the direct effect is negative, and larger in magnitude than the indirect effect. In this case, the proportion explained by mediation is negative. When the total effect is close to 0 (i.e., when the direct effect is

just slightly larger in magnitude than the indirect effect), the proportion explained by mediation is large in magnitude and negative.

Supplementary Table 1. Applied cancer anatomical site and morphology classification systems.

Anatomical tract	Malignancy	Anatomy: ICD 7[a]/8[b]	Morphology: CANC/24.1[c]	ICD-O-2[d]
Head and neck	Lip	1400-1409		
	Tongue	1410-1419		
	Salivary glands	1420-1429		
	Other oral cavity	143, 144, 148		
	Pharynx	145, 146, 147		
	Tonsils	1450		
Upper digestive tract	Esophagus adenocarcinoma	1500-1509	076, 096, 091	
	Esophagus squamous cell carcinoma	1500-1509	146, 166	
	Stomach	1510-1519		
	Liver primary	1550-1559, 156	886, 066	
	Biliary tract	1520, 1550-1559	196, 076	
	Pancreas	157, 1955		
Lower digestive tract	Small intestine	1520-1529		
	Colon	1530-1539		
	Rectum	1540-1548	076, 096, 091	
	Anus	1540-1548	146, 166	
Respiratory organs	Nasal cavity/sinuses	1600-1609		
	Larynx	161		
	Lung	162		
	Lung squamous cell	162		80703, 80833, 80823
	Lung adenocarcinoma	162		814, 825, 849, 848, 826
	Lung small cell carcinoma	162		8041, 8042, 8043, 8044, 8246
	Lung other non-small cell	162		
	Pleura mesothelioma	162	776	
Urinary organs	Urinary tract	1800-1819	111, 113, 114, 116	
	Kidney	1800-1809		
Skin, central nervous system, thyroid	Skin melanoma	1901-1919	176	
	Skin non-melanoma	1901-1919		
	Brain	1930, 1931, 1938, 1939	026, 406, 416, 436, 456, 476, 486	
	Meninges	1930-1939	461, 466, 511, 531, 533, 536	
	Thyroid well-differentiated	194	076, 096, 091	
	Thyroid anaplastic	194	046, 196, 186 or 446	
Hematological malignancies	Non-Hodgkin lymphoma	2000-2024		
	Chronic lymphocytic leukemia[b]	2041		
	Hodgkin lymphoma	201		
	Multiple myeloma	203	336	
	Acute lymphocytic leukemia[b]	2040, 2049		
	Acute myeloid leukemia[b]	2050, 2060, 2072, 2073		
	Chronic myeloid leukemia[b]	2051		

[a]The International Classification of Diseases version 7 (ICD-7)

[b]The International Classification of Diseases version 8 (ICD-8, 1980-)

[c]The World Health Organization Histological Classification of Neoplasms (WHO/HS/CANC/24.1)

[d]The International Classification of Diseases for Oncology, second edition (ICD-O-2, 1993-)

Supplementary Figure 1. Proportion mediated as a function of the total effect.

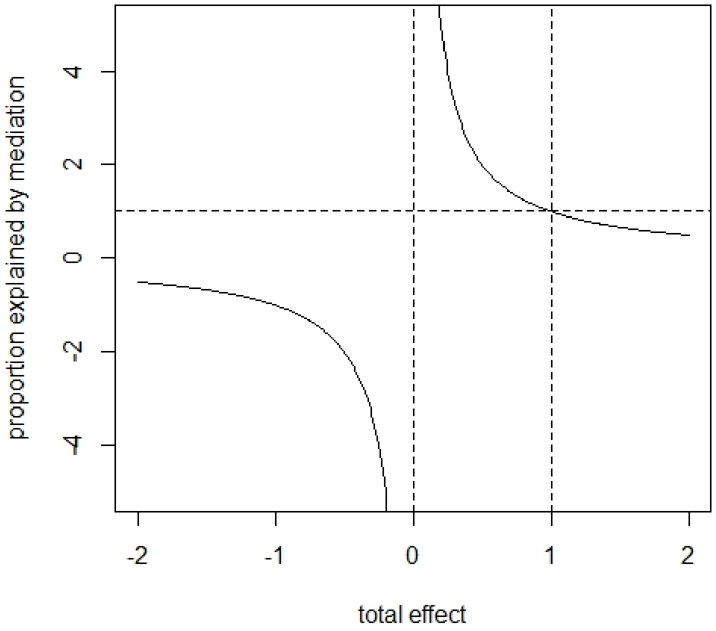

Supplementary Figure 2. Schoenfeld residuals over log-transformed survival time for female (blue) and male (red) patients. Parallel curves indicate that the proportional hazards assumption is met for sex; deviations suggest potential violations. Residuals are shown for all 28 cancer sites included in mediation analysis.

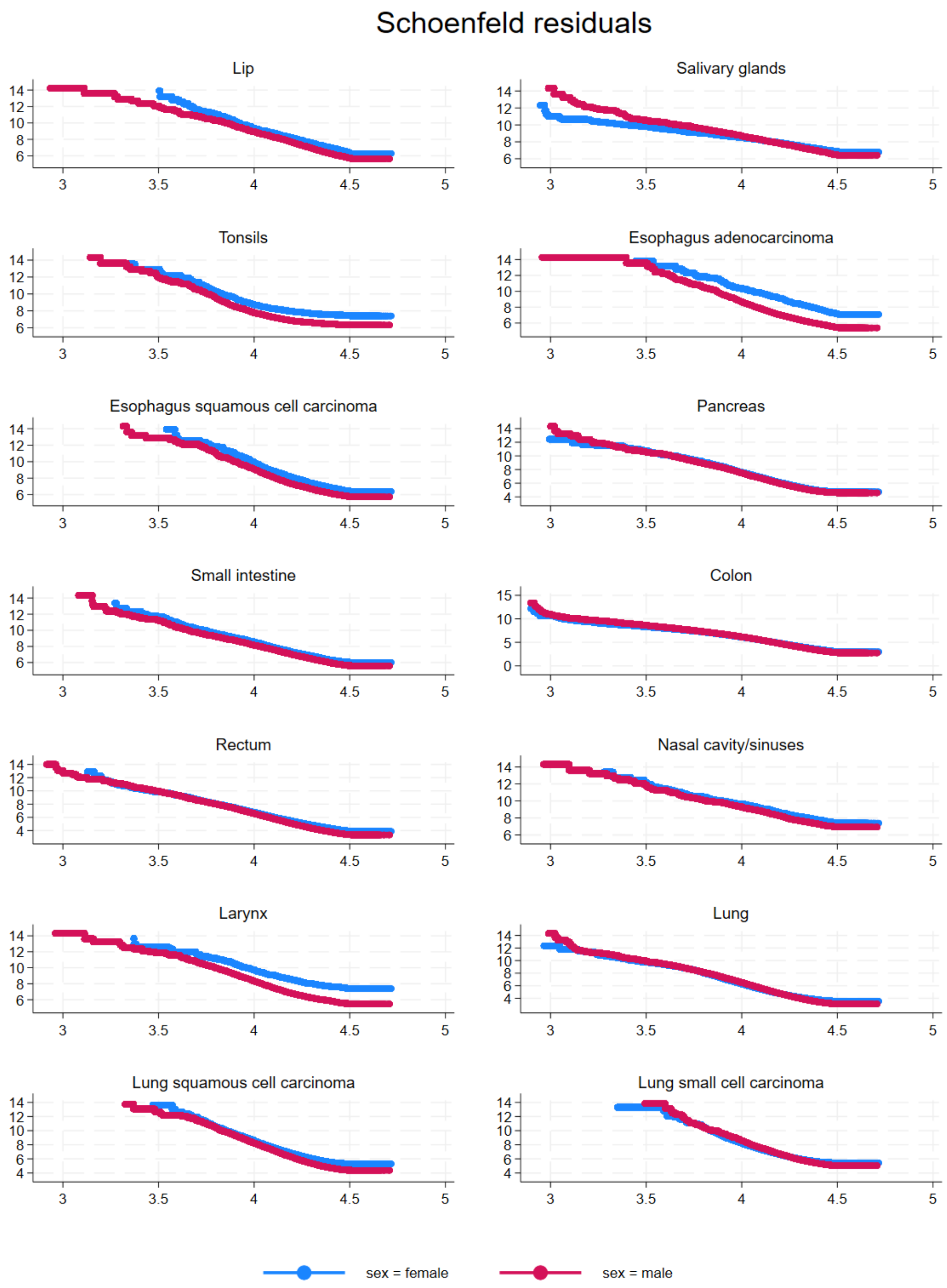

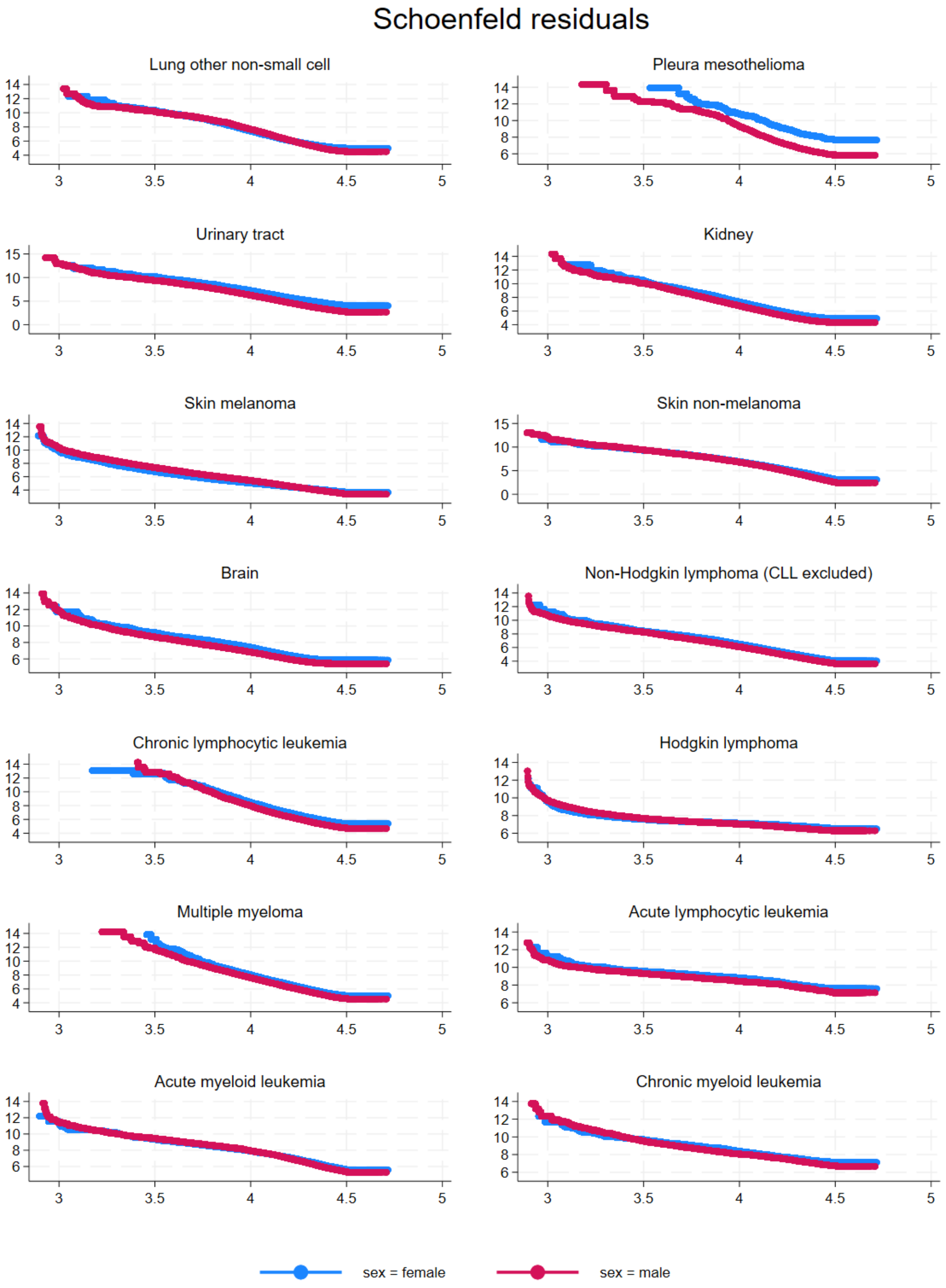
Schoenfeld residuals
Lung other non-small cell
Pleura mesothelioma
Urinary tract
Kidney
Skin melanoma
Skin non-melanoma
Brain
Non-Hodgkin lymphoma (CLL excluded)
Chronic lymphocytic leukemia
Hodgkin lymphoma
Multiple myeloma
Acute lymphocytic leukemia
Acute myeloid leukemia
Chronic myeloid leukemia
sex = female
sex = male

Supplementary Figure 3. Mean attained (adult) body height in cm in Swedish women and men over year of birth.

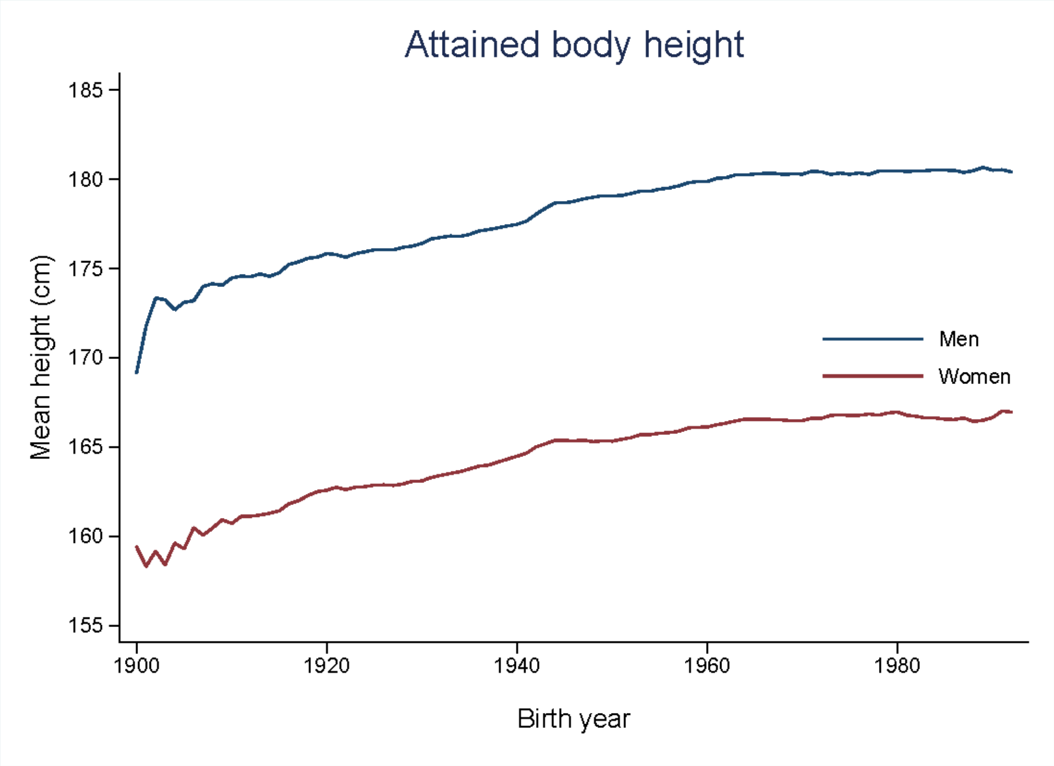

Supplementary references

1. Robins JM, Greenland S. Identifiability and exchangeability for direct and indirect effects. *Epidemiology (Cambridge, Mass)* 1992;**3**: 143-55.

2. Proceedings of the 17th Conference in Uncertainty in Artificial Intelligence, Jack SB, Daphne K, eds.2001.

3. VanderWeele T. *Explanation in Causal Inference*ed.: Oxford University Press Inc, 2015.

4. Nevo D, Liao X, Spiegelman D. Estimation and Inference for the Mediation Proportion. *The international journal of biostatistics* 2017;**13**.

5. Hafeman DM. "Proportion explained": a causal interpretation for standard measures of indirect effect? *American journal of epidemiology* 2009;**170**: 1443-8.

6. Sjölander A. An Approximate Expression for the Proportion Explained by Mediation in Survival Analysis. *Epidemiology (Cambridge, Mass)* 2020;**31**: e21-e2.
